# Use of food labels by adolescents to make healthier choices on snacks: a cross-sectional study from Sri Lanka

**DOI:** 10.1186/s12889-016-3422-1

**Published:** 2016-08-08

**Authors:** Ishanka A. Talagala, Carukshi Arambepola

**Affiliations:** 1 Management, Development and Planning Unit, Ministry of Health, Nutrition & Indigenous Medicine, Colombo 8, Sri Lanka; 2 Faculty of Medicine, University of Colombo, Kynsey Road, Colombo 8, Sri Lanka

**Keywords:** Snacking, Food labels, Adolescents, Marketing strategies

## Abstract

**Background:**

Unhealthy snacking is commonly seen among adolescents. Therefore, use of food labels is promoted for making healthier choices on packaged snacks. This study was conducted to assess the use of food labels in making choices on packaged snack and its associated factors among adolescents.

**Methods:**

A cross–sectional study was conducted in 2012 among 542 Grade 12 students in Sri Lanka. Eight classes were selected as ‘clusters’ for the study (two classes each from two schools that were selected randomly from each list of ‘Girls only’ and ‘Boys only’ schools in Colombo district). A self-administered questionnaire assessed their socio-demography, snacking behaviour, attitudes and nutrition knowledge related to food labels. Adolescents’ use of labels was assessed by three practices (label reading frequency, attention paid to label contents and correct interpretation of six hypothetical labels of snacks). Based on total scores obtained for the three practices, ‘satisfactory’ (score ≥75^th^ percentile mark) and ‘unsatisfactory’ (score <75^th^ percentile mark) label users were identified. Using SPSS, associations were assessed at 0.05 significance level using Chi-square-test.

**Results:**

Of the participants, 51 % were males; 61 % spent their pocket money at least once/week on packaged snacks; predominantly on biscuits (85 %) and cola-drinks (77 %) and 88 % selected snacks on their own. The majority (74.5 %) was frequent (‘always’ or ‘most often’) label readers with female predominance (*p* < 0.05). Over 74 % paid attention frequently to the brand name (75 %), price (85 %) and nutrition panel (81 %). Over 64 % were able to select the better food label when given a choice between two snacks, although some did it for reasons such as attractive label (63 %). The majority (84 %) had good knowledge (obtaining more than the 75^th^ percentile mark) on interpreting labels.

Although not statistically significant, ‘unsatisfactory’ label use was higher among males (73 %), purchasing power (70.4 %) and unhealthy snacking behaviour (73 %). In contrast, among the marketing strategies, identifying known brands (73.2 %) and imported products (75.8 %) as ‘good’ products were significantly associated with ‘unsatisfactory’ label use (*p* < 0.05).

**Conclusions:**

Despite having good knowledge and positive attitudes, food label use is unsatisfactory among adolescents. Skills in reading labels should be addressed in the ‘School canteen policy’ in Sri Lanka.

## Background

Urbanization has led to a dramatic shift in the food consumption patterns around the world [1]. It has increased the availability of unhealthy food, which contributes substantially as a risk factor to the pandemic of non-communicable diseases (NCDs) along with other lifestyle related risk factors such as physical inactivity, consumption of alcohol and smoking. Though seen mostly during adult life, diet-related NCDs result from unhealthy dietary practices acquired since childhood [2]. Therefore, healthy eating behaviour should be established early in life as a strategy to prevent NCDs [3].

Snacks are light quick meals consumed between main meals [4] and include pre-cooked and ready-to-eat food items, and non-alcoholic beverages. In the United States (US), snacking has become a popular eating habit across all age groups and in particular, among adolescents aged 10–19 years, with its prevalence ranging between 60 and 98 % [5]. Snacking among adolescents is higher compared to other age-groups in response to their growth spurt to fulfil their hunger gaps and nutritional demands. In addition, their snacking tendency is heavily influenced by the quest for independence, peer acceptance, self-image and mood [6,7]. Over the past few decades, the contribution of snacks to daily energy intake has been amplified from 20 to 23 % among young adults [8]. They have shown a strong inclination towards packaged snacks that are high in fat, salt, sugar and calories [9] thus, resulting in NCDs in their adult life.

Since there are many healthy options available among snacks, snacking can be made part of a healthy diet especially among adolescents. This requires empowering them to seek accurate and reliable information on healthy snacks, to decide which snack is healthy and which is not. In this regard, labels on packaged snacks serve as a reliable source of nutrition-related information. These labels provide simple visual guides that enable the consumers to make healthier choices at a glance [10]. The consumers are expected to translate the information given on labels of packaged snacks into healthy choices at the point of purchase [11]. This involves the practices on the frequency of reading labels, attention paid to the contents of a label and interpretation of the information given on labels for making a healthy choice.

It is shown that nutrition education, age, sex and attitudes predict the food label use by the adolescents [12]. Adolescents are specifically targeted to intense marketing efforts by manufacturing companies to promote unhealthy snacks, since they represent the future adult consumers [7]. Few examples of these marketing strategies include portrayal of popular figures, brand promotion and false health, nutritional or ethical claims displayed on food/drink labels. For minimizing such vulnerabilities, adolescents should be well-equipped with correct knowledge and attitudes on the use of food labels [13].

Despite its importance, understanding the use of food labels by adolescents to choose healthier snacks is limited [10]. Further, other than a few studies conducted in developed countries that show lack of knowledge and skills of adolescents in interpreting food labels [14], scarce evidence is available from the developing countries. In Sri Lanka—a country in South Asia, food labelling is mandatory by law for any packaged/bottled food/drink item according to the Food Act, 1980 [15], while there are food labelling and advertising regulations to which the manufacturers should abide by [16]. This study aimed to examine the use of food labels in making choices on packaged snacks/drinks and its associated factors among adolescents in Sri Lanka.

## Methods

A school-based cross-sectional study was conducted in 2012 among students aged 16–17 years living in the district of Colombo, Sri Lanka. This district is the commercial capital of Sri Lanka and consists of 403 schools, of which 68 schools have at least two Grade 12 classes in all Science, Mathematics, Commerce and Arts subject streams (Type I AB) (Personal communication with the Education department, Colombo district, Sri Lanka: School census 01/06/2011). A three-stage, stratified cluster sampling method was used to select the sample. Initially, the schools were stratified into ‘Girls only’ and ‘Boys only’ schools of Type I AB. Then, two schools from each stratified list were randomly selected. Thereafter, two Grade 12 classes were selected randomly from each selected school as clusters. Finally, all students in each selected class were recruited for the study. Students who were on medically recommended specific diet plans with restrictions for snacking were excluded from the study. The calculated sample size was 524 based on 0.05 precision, 50 % expected proportion of satisfactory food label users, 1.3 design effect (to overcome the loss of effectiveness by the use of cluster sampling, instead of simple random sampling) and 5 % non-response. Since students were recruited as clusters of classes, a total of 542 students were finally included in the study.

Prior to data collection, informed written consent was obtained from the parent/guardian of each eligible student and also from the students through an assent form. Administrative clearance was obtained from the Director of Education—Western Province and from the principals of each selected school. Ethics clearance was obtained from the Ethics Review Committee of the Faculty of Medicine, University of Colombo.

A self-administered questionnaire prepared in local languages was used for collecting data on socio-demography, snacking behaviour, the use of food labels when making choices on packaged snacks, attitudes and nutrition knowledge related to food label use among the students. A packaged snack was defined as ‘a small meal or amount of food/drink in a packaged/bottled form that is usually taken between main meals’ [4]. A food/drink label was defined as ‘a visual guide attached to a packaged/bottled food/drink item, giving the basic information about the product’ [15].

**Fig. 1:**
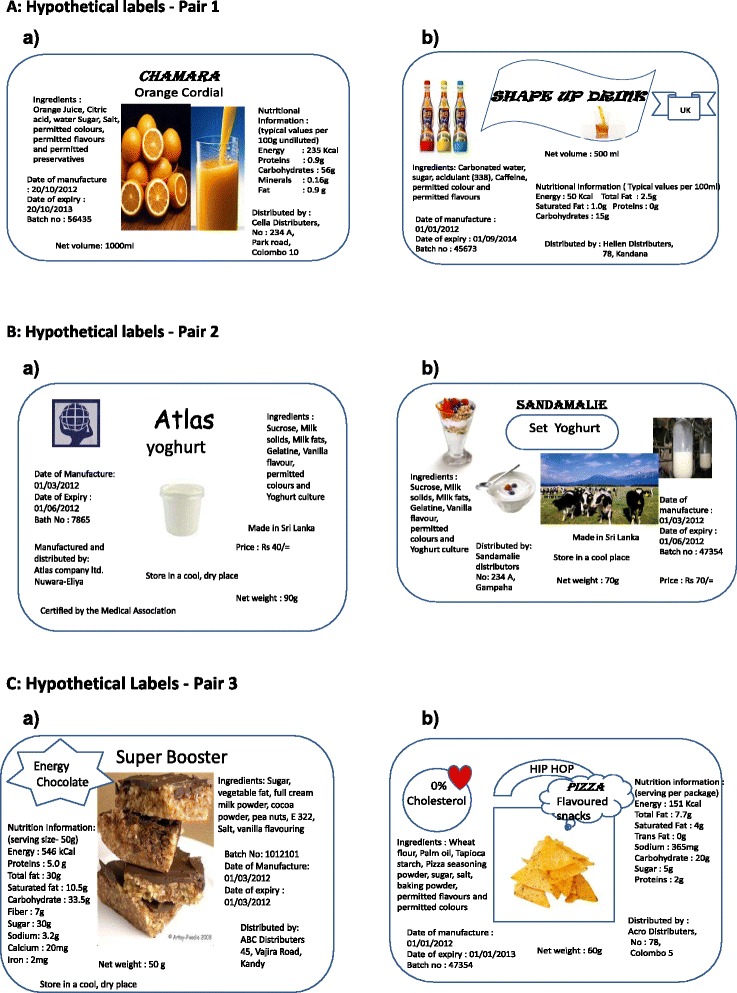
Hypothetical labels created by the authors to collect information on the label use by the student

The use of labels by adolescents was determined by the following three practices: 1) frequency of reading labels, 2) attention paid to label contents and 3) interpretation of the information given on labels for making a healthy choice. For easy recall, the frequency of label reading (the first practice) was assessed by inquiring the usual number of labels that each student would read for every ten snacks they consume, and graded as: ‘always’ (reading all ten labels), ‘most of the time’ (reading 6–9 labels), ‘sometimes’ (reading 1–5 labels) or ‘not at all’ (reading none). The second practice was assessed by asking the students to grade the contents of a label based on the attention that they usually pay on purchasing a snack. The grading system was the same as in the first practice. The third practice was assessed by displaying three pairs of hypothetical labels (each pair on a similar type of snack) and asking the participants to select the healthier option from each pair based on the information given on the label and to state the main reason for their selection. For this purpose, the authors created labels similar to the products available in the market as they believed that it was unethical to use the actual labels of snacks that were currently available in the market (Fig. 1), which could indirectly result in brand promotion among school children.

### Classification of ‘satisfactory’ and ‘unsatisfactory’ users of food labels

Based on the marking scheme and the cut off values developed by an expert panel of nutritionists, marks were awarded to each practice related to the use of labels: 0–5 marks for the frequency of reading labels, 0–24 marks for attention paid to the contents of food/drink label, and -6 to +27 marks for the interpretation of hypothetical food labels (-2 to +9 marks for each pair). Marks were added to obtain the total score (range: from -6 to 56) obtained by each student on the use of labels. In the frequency distribution of these scores, score of 30 marked the 75^th^ percentile, which was taken as the cut-off to group the students into ‘satisfactory label users’ (≥30 marks) and ‘unsatisfactory label users’ (<30 marks).

### Classification of ‘good’ and ‘poor’ nutrition related knowledge of students

There were 10 questions to assess the nutrition related knowledge of the students in the questionnaire. Based on the marking scheme and the cut off values developed by the panel of nutritionists, the correct answer given for each question in the knowledge questionnaire was awarded one mark (marks range: 0–10). In the frequency distribution of total marks obtained by students, the 75^th^ percentile was at 8 marks, which was taken as the cut-off to group students into ‘good knowledge’ (≥8 marks) and ‘poor knowledge’ (< 8 marks) categories.

The students’ attitudes were assessed on the marketing strategies used by the manufacturers for promoting unhealthy snacks. Questions on knowledge related to nutrition, basic contents on food labels and the hypothetical labels created were all based on the guidelines and regulations on labelling and advertising given in the Food Act, 1980 in Sri Lanka [15].

#### Data analysis

SPSS (version 20, 2011, IBM Corp., USA) was used for data analysis. Descriptive statistics including mean (SD) for continuous data and proportions for categorical data were used. The factors associated with ‘unsatisfactory’ label use of the students were assessed using Chi-square test at the 0.05 significance level.

## Results

A total of 542 students participated in the study. They belonged to Bio-science (20.8 %), Mathematics (22.5 %), Commerce (30.3 %) and Arts (26.4 %) streams. Of them, 51.1 % were male students. All participants snacked on packaged food and drinks. They spent pocket money received from their parents/guardians on food/drinks (98.9 % females, 97.1 % males). Their snacking behaviour is shown in Table 1. The most frequently consumed packaged food snacks were biscuits (85.4 %), instant noodles (66 %) and chocolates (61 %), while the bottled/packaged drinks were colas (76.9 %), milk (70.7 %) and fruit juices (26.4 %).

**Table 1:** Snacking behaviour of adolescents in Grade 12 (*n* = 542)

Snacking behaviour	Number	Percentage
Frequency of snacking		
More than 5 times a week	70	12.9
3–5 times a week	197	36.3
1–2 times a week	275	50.7
Most frequent snacking time^a^		
During school interval	209	40.9
During tuition classes outside school	126	24.7
At home	74	14.5
During school extra-curricular activities	65	12.7
During recreational activities outside school	37	7.2
Snacking with peers/siblings^b^		
Yes	425	80.0
No*	106	20.0
Decision made on the snack		
Alone	479	88.4
Parents	36	6.6
Peer/siblings	27	5.0
Most commonly consumed packaged foodsnack^c^		
Biscuits	463	85.4
Noodles	359	66.2
Chocolates	332	61.3
Most commonly consumed packaged/bottled drink snack^c^		
Cola drinks	417	76.9
Milk	383	70.7

^a^Information was not available for 31 students

^b^Information was not available for 11 students

^c^Students were asked to write three most commonly consumed packaged food and drink items

*With parents/alone

### Practices of students in relation to their use of food labels

1. Frequency of reading labels

With the exception of 9 students (1.7 %), all reported that they read labels when consuming a packaged snack. Of those students, 74.6 % were ‘frequent’ (those who read labels ‘always’ or ‘most of the time’) label readers (Table 2). Compared to males (70 %), a significantly higher proportion of females (79.2 %) were ‘frequent’ label readers (*p* = 0.01).

**Table 2:** Practices on the label reading frequency and attention paid to label contents among adolescents

Practices	Always	Most	Sometimes	Not at all
	%	of the time %	%	%
Frequency of reading labels^a^	40.8	33.8	23.7	1.7
Attention paid to contents^b^				
Brand name	24.6	50.1	20.5	4.9
Price	34.7	50.3	12.0	3.0
Expiry date	93.6	5.4	0.4	0.6
Manufactured date	55.2	26.6	13.7	4.5
Serving amount	7.5	22.8	47.5	22.2
Batch number	2.6	8.7	32	56.7
Nutrition information	38.4	43.7	13.2	4.7
Country of manufacture	9.2	34.7	37.8	18.3
Storage instructions	21.5	41.7	21.3	15.5
Medical certification	28.8	38.0	20.2	13.0
Attractiveness of the label	6.2	22.4	33.7	37.7
Readability of the label	30.3	41.8	20.5	7.4

^a^ *N* = 542 (the total number of participants who responded)

^b^ *N* = 533 (out of the total number of participants responded, 9 students who never read food labels were excluded)

2. Attention paid to the label contents

When consuming a packaged snack for the first time, more than 70 % of the students paid attention ‘always’ or ‘most of the time’ to the expiry date, manufactured date, price, brand name, nutrition panel and the readability of the label (Table 2).

3. Interpreting the information on labels for making a healthy choice

When three pairs of labels were given to students for their interpretation on healthier choices, over 65 % of them were able to select the healthier option. However, the main reasons the students have given for their choices showed a wide variation (Table 3).

**Table 3:** Interpretation of labels by the grade 12 adolescents for their selection of healthier snacks

Option selected based on each pair of hypothetical labels^a^	Reasons for selection
Pair 1 (Fig. 1a)	
Healthier option - 85 % (*n* = 453)	• Natural ingredients (32 %)
(Natural fruit drink—local product with a picture of oranges, a true claim on ‘no added sugar’)	• High nutritive value (23 %)
	• Local product (ethical claim) (6 %)
	• Fruits showing ‘healthiness’ (4 %)
Less healthy option - 15 % (*n* = 80)	• Imported product (26 %)
(Fizzy drink—imported product with an eye-catching picture, a false claim on ‘empty calories’)	• Attractive label (16 %)
Pair 2 (Fig. 1b)	
Healthier option - 70.5 % (*n* = 376)	• Medical recommendation (73 %)
(Ordinary label—same nutrients, low price, a claim on ‘Certified by the Medical Association’)	• Reasonable price (15 %)
Less healthy option-29.5 % (*n* = 157)	• Attractive label (63 %)
(Attractive label—same nutrients, high price, no claims)	• Costly, so better quality (2.5 %)
Pair 3 (Fig. 1c)	
Healthier option - 64.5 % (*n* = 344)	• Taste of chocolate (35 %)
(High-energy product—high calories and all major nutrients included in the nutrition panel, no claims)	• High calories (21 %)
	• Nutritive value (16 %)
	• Attractive label (11 %)
Less healthy option -35.5 % (*n* = 189)	• Zero cholesterol (65 %)
(Less energy product—false claim on ‘zero cholesterol’ despite saturated fatty acids in the nutrition panel)	• Pizza like taste (15 %)
	• Quick snack (7 %)

^a^ *N* = 533 (9 students who never read food labels were excluded)

The total score obtained by the students for their overall use of food labels based on the three practices of frequency of reading labels, attention paid to the label contents and interpreting the information on labels for making a healthy choice ranged from -1 to +45 (mean score =23.9; SD = 9.1). When categorised based on 30 mark as the cut-off, 70 % of the adolescents were identified as ‘unsatisfactory’ users of food labels.

### Nutrition related knowledge required for use of labels

‘Good’ nutrition related knowledge was demonstrated by 84.1 % of students. The majority knew how to interpret the “best before date” and “high in fats” terms, fibre content, and the use of preservatives, colours and additives (Table 4). However, their interpretation of % Recommended Daily Allowance (% RDA) and “natural product” was relatively poor.

**Table 4:** Nutrition related knowledge of grade 12 adolescents required for reading labels on packaged snacks (*n* = 533)^a^

Knowledge domain	Students with good knowledge	
	No.	%
Interpretation of “best before date” on the label	471	88.5 %
Interpretation of “high in fats” on the label	434	81.7 %
Use of permitted preservatives in snacks	470	88.5 %
Fibre content in snacks	360	67.8 %
Use of permitted colours and additives in snacks	281	52.9 %
Unhealthy fat types in snacks	252	47.5 %
Interpretation of serving size	274	51.6 %
Definition of % RDA	143	26.9 %
Interpretation of "natural product” on the label	52	9.8 %
Overall ‘Good’ knowledge	448	84.1 %

^a^Of the 542 in the sample, 9 students who never read labels were excluded

### Attitudes on the marketing strategies used to promote snacks

The majority of students ‘agreed’ or ‘strongly agreed’ that information given on the labels of packaged snacks were trustworthy if it has a brand name that is popular or the product was ‘expensive’ (Table 5). They did not have a similar opinion for snacks that were frequently advertised on television or marketed as ‘imported’, endorsed by a celebrity or its ‘healthiness’ portrayed by a sports star.

**Table 5:** Attitudes of the students towards the marketing strategies used by the manufacturers to promote unhealthy snacks (*n* = 533)^a^

Information given on labels of packaged snacks is trustworthy, if	Strongly agree	Agree	Disagree	Strongly disagree
	%	%	%	%
- Has a popular brand name	15.0	48.8	30.2	6.0
- Advertised frequently on media	3.9	5.9	66.6	23.6
- Marketed as ‘imported’	9.2	28.0	49.5	13.3
- Marketed as ‘expensive’	10.5	54.6	28.1	6.8
- Endorsed by a celebrity	6.2	3.5	41.1	49.2
- A sports star is portrayed	4.1	7.1	51.4	37.4

^a^Of the 542 in the sample, 9 students were excluded as they did not read labels at all

### Factors associated with ‘unsatisfactory’ use of labels on packaged snacks

‘Unsatisfactory’ use of labels on packaged snacks was higher among adolescents who were males (73.3 % versus 66.5 % females), in Science subject stream (70.4 % versus 69.7 % non-Science), snacking >3 times/week (72.7 % versus 67 % ≤ 3 times/week) and snacking with parents/alone (70.2 % versus 69.9 % with peers/siblings), and making their own decisions on snacks (71.2 % versus 60.7 % decision made by others). These differences were not statistically significant (*p* > 0.05), implying that the snacking behaviour of adolescents is not associated with their label use.

With regards to nutrition related knowledge, ‘unsatisfactory’ use of labels was almost the same among students having ‘poor’ nutrition knowledge and those having a ‘good’ knowledge (70.6 % versus 69.9 %). However, specifically, the knowledge of unsatisfactory label users was ‘poor’ in interpreting the ‘best before’ date (73.8 % versus 61.9 %), % RDA (70.4 % versus 68.5 %), preservatives (70.5 % versus 69.8 %), number of servings (70.8 % versus 69.0 %) and the fibre content (72.5 % versus 68.6 %) given on a packaged snack. However, none of these differences were statistically significant (*p* > 0.05).

With regards to attitudes on marketing strategies used by the manufacturers, unsatisfactory label use was significantly higher (*p* < 0.01) among adolescents who considered information given on labels to be trustworthy, if the brand name was popular (73.2 % versus 64.2 %) or marketed as an ‘imported’ product (75.8 % versus 66.6 %). No other attitude was significant (Table 6).

**Table 6:** Comparison of unsatisfactory use of food labels by their nutrition related knowledge and attitudes towards marketing strategies used for promoting snacks (*n* = 533)^a^

Characteristic	Total No.	Unsatisfactory label users		Satisfactory label users		Level of Significance^b^
		No.	%	No.	%	
Nutrition related knowledge						
Good 448	448	313	69.9	135	30.1	*p* =0.89
Poor 85	85	60	70.6	25	29.4	
Attitudes						
Brand name						***p*** **= 0.03**
Disagree	193	124	64.2	69	35.8	
Agree	340	249	73.2	91	26.8	
Television advertisements						*p* = 0.25
Disagree	481	333	69.2	148	30.8	
Agree	52	40	76.9	12	23.1	
Imported products						***p*** **= 0.025**
Disagree	335	223	66.6	112	33.4	
Agree	198	150	75.8	48	24.2	
Portrayal of a sports star						*p* = 0.55
Disagree	473	333	70.4	140	29.6	
Agree	60	40	66.7	20	33.3	
Endorsement of a celebrity						*p* = 0.07
Disagree	481	331	68.8	150	31.2	
Agree	52	42	80.8	10	19.2	
Expensive products						*p* = 0.41
Disagree	186	126	67.7	60	32.3	
Agree	347	247	71.2	100	28.8	

^a^Of the 542 in the sample, 9 students were excluded as they did not read labels at all

^b^Significant associations (significant level *p* < 0.05) shown in bold letters

## Discussion

Our study highlights the unsatisfactory use of food labels on packaged snacks by adolescents. Despite reading labels ‘frequently’ (always or most of the time) and paying attention to a variety of contents in the label, their interpretation of the information given on labels for making a healthy snack choice was grossly inadequate. Their decisions on packaged snacks were mostly driven by false claims given on labels, rather than by the nutritional value of the product. Despite the majority of the students having ‘good’ nutrition related knowledge, they failed in translating it into practice when making decisions on food/drink snacks. Marketing strategies that significantly associated with unsatisfactory label use were on brand promotion and imported products.

A study in the US found that teenagers usually spend 18 % of their money on food [17]. According to our study, Sri Lankan adolescents too were no different. However, they retained their autonomy when making decisions on snacks. This is in contrast to the peer influence commonly seen among adolescents. A survey conducted among 10–16 year old adolescents in Denmark showed that adolescents purchased and consumed snacks that support their self-esteem when socializing with peers [18].

In our study, despite the availability of healthier snacking options, they showed a tendency to select unhealthy ones such as cola drinks (77 %). Similarly, a study among public high school students in North Los Angeles County found that 50.1 % were drinking two Soda glasses or more per day during the past 1 year [19]. Adolescents are well known for exploring new tastes and trends in what they eat and drink. As a result, they are increasingly adopting unhealthy dietary practices especially in relation to their energy consumption, ultimately resulting in obesity and other related NCDs. It is shown that among the middle school students in Minneapolis-St Paul metropolitan area, frequent snacking with high calories but low in nutrition food was adversely associated with their body mass index [20]. The World Health Organization (WHO) states that early lesions of atherosclerosis are found in obese children [21]. Primordial prevention efforts should therefore be directed towards discouraging adolescents from adopting harmful dietary practices. Improving their skills towards smart shopping through food labels would be one such effort to re-shape them to adopt healthy choices.

In our study, we determined the unsatisfactory use of labels based on three practices, of which one was interpretation of the information given on labels. For this purpose, most previous studies have used self-administered questionnaires: paper based/web based [22] and observations made in a cafeteria [23] to assess adolescents’ use of already known food labels. Using a questionnaire could simply underestimate or overestimate their actual consumption owing to recall bias, while direct observations may prompt them to deviate from their usual behaviour. We could overcome these issues in our study by using hypothetical labels to assess how adolescents would apply their knowledge and skills on labels for making a healthy snack choice. Since students relied only on the facts given on the hypothetical label to make their choice, the information obtained was independent of their peer and parental influences, as well as their previous experience with actual products (eg: taste).

Frequent label reading is a good practice that needs to be inculcated in an individual from adolescence. However, the majority of studies have shown that adolescents read food labels less frequently. According to the National Health and Nutrition Examination Survey 2005-6 in USA, less than 25 % of adolescents used food labels during food purchase [14], while it was 37.8 % among female university students in Korea [24] and 31.3 % in another study among students in Louisiana [25]. In contrast, one study conducted in Kolkata, India among schooling adolescents showed that 88 % of them read food labels [26]. In comparison, the majority in our study read labels ‘always’ or ‘most of the time’. This highlights the health conscious nature of today’s youth. In our study, females were seen to be more frequent label readers than males. This finding is in line with many other studies conducted in developed countries [27–29]. Since females are the grocery shoppers and meal planners of households in Sri Lanka, improving their skills on reading labels is particularly of value.

Our study shows that adolescents were selective when reading labels. They seemed to pay more attention to the expiry date and manufactured date than to the nutrition panel. This reflects their health concerns—acute illnesses such as diarrhoea taking priority over chronic illnesses such as NCDs. It has also been shown that general awareness among school children regarding lifestyle related risk factors of NCDs is unsatisfactory [30], which needs to be addressed in the school curriculum. Our study further shows that adolescents pay more attention to the price on food labels. A randomized controlled trial conducted in high schools in Netherlands also found that students make healthier choices with the availability of such options, combined with labelling and reduced price [31]. Unfortunately in developing countries, adolescents living in urban cities are exposed to a wide range of unhealthy packaged snacks that are usually much cheaper than the healthier options [32].

Over 70 % of the adolescents in our study were ‘unsatisfactory’ users of food labels. Incorrect interpretation of the healthiness of snack based on the label was the main contributor towards this use. Wojcicki et al (2012) have shown that having inadequate nutritional knowledge would prompt adolescents to misinterpret its healthiness [14]. However, this was unlikely in our study, as the majority of students showed good knowledge on nutrition. Furthermore, we could not elicit a significant association between poor knowledge and unsatisfactory label use. Studies done in Australia and US have shown discrepancies between the adolescents’ nutritional knowledge and dietary behaviour [11,33]. This highlights that improving adolescents’ nutrition related knowledge alone would not be effective, but finding reasons as to why such knowledge is not applied by them on labels through means of qualitative research by means of qualitative research, would be of practical importance.

Food labels help to improve the credibility of nutrition information given on packaged snacks [34]. Thus, food regulations in most countries, including Sri Lanka enforce mandatory labelling for packaged products, resulting in several positive outcomes on changed consumer behaviour [15,29]. However, adolescents prefer to make decisions on snacks quickly at a glance, thus reading long lists of nutrients given on labels would become rather cumbersome [35]. According to the Pan-European project, consumers seemed to pay attention to the nutritional label for an average of 25–100 milliseconds, which is grossly inadequate to understand the information given [36]. In this backdrop, traffic light labelling of nutrients in packaged snacks would serve as a simple tool for making a healthy choice [36]. The relative healthiness of a product is identified by bench-marking the nutrients and colour-coding each as green, amber and red using nutrient profiling [37]. When the front-of-pack Guideline Daily Amounts was coupled with the traffic light system, Spanish secondary school adolescents aged 14–16 years significantly seemed to choose healthier options than with monochrome Guideline Daily Amounts [38].

The majority in our study was able to select the healthier option when given the hypothetical food labels, but did so for various reasons such as their previous pleasant/unpleasant experience of a similar product, imported product, attractiveness of the label, and false nutritional and health claims given on food labels, rather than by correctly interpreting the label. Specifically, false claims affect food choices as consumers seem to value the products with claims [29]. In recognizing this deficiency, Codex alimentarius defines specific health related information on labels that are regulated by law towards consumer protection. One such aspect is on claims. Developed countries have used nutrient profiling to provide scientific evidence to regulate nutritional claims on snacks. For example, it has been the basis for banning some of the packaged snacks with false claims from prime time television that target children [39]. Until such regulations are in place in developing countries, adolescents should at least be empowered to compare the claims given with its nutrition panel.

Another source of misinformation is due to poor identification of the marketing strategies used by manufacturers for attracting adolescents. These include endorsement and portrayal of celebrities, medical/health related organizations and sport stars to depict its ‘healthiness’. Our study could not elicit a significant association of unsatisfactory use of labels with these typical marketing strategies. Instead, the negative attitudes associated with popular brand names and imported products marketed showed a significant relationship with unsatisfactory label use. In addition, 95 % of the students in our study paid attention to the brand name among other contents of a label. This could be a result of the effects of print and visual media on promoting different brands as well as due to the practice of brand shopping by the parents.

Brand name plays a major role in the decision of snacks. Adolescents grow in a media saturated environment with food marketers targeting adolescents as their future adult consumers [7] and they have become the primary target for digital marketing [40]. Marketers of fast food, snack food and soft drinks, use various digital marketing techniques to target adolescents [41] and they are approached via subtle methods intending to influence brand awareness, brand preference and brand loyalty [7] thereby, influencing the behavioural practices of young people, bypassing the rational decision making. Brand preference can be easily inculcated among adolescents by intense marketing efforts with special emphasis on the youth [7,42]. Brand shopping could easily truncate their nutritional search and undermine other efforts of teaching healthy eating practices to adolescents, thus should be addressed promptly.

Ministry of health and the Ministry of Education in Sri Lanka are in collaboration with improving healthy dietary habits among adolescents through the launching of ‘School canteen policy’. It prohibits the sale of carbonated fizzy drinks, sweetened drinks, and high fat, salt and sugar food items in schools and promotes the sale of fruits, grain products and liquid milk [43]. As such, improving the use of food labels via school health promotion programme and the school canteen policy are recommended for empowering adolescents to select healthier snacks.

### Limitations of the study

In this study, data collection was done through a self-administered questionnaire which might have resulted in misinterpretation of some questions and misreporting. Also, their snacking behaviour and the general use of food labels was assessed retrospectively. Both would have associated with recall bias. Most of the factors underlying the students’ use of food/drink labels are inter-related, thus a logistic regression model would reveal associations better adjusted by confounders. Generalizability of the findings is for Grade 12 students in the district of Colombo.

## Conclusions

The findings show that despite having a good nutrition related knowledge, the use of food/drink labels among adolescents is unsatisfactory. This emphasizes the need to develop and implement programmes aimed at adolescents through the existing school health promotion programme to improve the usage of food labels on making healthy choices on their food/drink snacks. Programmes should be implemented at school level to transform the attitudes of students on brand promotion to overrule the adverse marketing efforts aimed at adolescents. In addition, improving the skills of interpreting false nutritional and health claims on labels would be essential.

## Abbreviations

% RDA, % Recommended Daily Allowance; NCDs, non communicable diseases; US, United States; WHO, World Health Organization
